# Efficacy of a mobile application for smoking cessation in young people: study protocol for a clustered, randomized trial

**DOI:** 10.1186/1471-2458-13-704

**Published:** 2013-08-01

**Authors:** Empar Valdivieso-López, Gemma Flores-Mateo, Juan-Domingo Molina-Gómez, Cristina Rey-Reñones, María-Luisa Barrera Uriarte, Jordi Duch, Araceli Valverde

**Affiliations:** 1 Centre d’Atenció Primària Bonavista, Direcció d’Atenció Primària Tarragona, Institut Català de la Salut, Tarragona, Spain; 2 Unitat de Suport a la Recerca Tarragona - Reus, Institut Universitari d’Investigació en Atenció Primària Jordi Gol (IDIAP Jordi Gol), Tarragona, Spain; 3 Departamento Informática Primaria Camp de Tarragona, Institut Català de la Salut, Tarragona, Spain; 4 Centre d’Atenció Primària Llibertat, Direcció d’Atenció Primària Tarragona, Institut Català de la Salut, Reus, Spain; 5 Centre d’Atenció Primària Torreforta - La Granja, Direcció d’Atenció Primària Tarragona, Institut Català de la Salut, Tarragona, Spain; 6 Departament d’Enginyeria Informàtica i Matemàtiques, Universitat Rovira i Virgili, Tarragona, Spain; 7 Agència de Salut Pública de Catalunya. Departament de Salut. Generalitat de Catalunya, Barcelona, Spain

**Keywords:** Randomized controlled trial, Smoking cessation, Technology, Primary health care

## Abstract

**Background:**

Tobacco consumption is the most preventable cause of morbidity-mortality in the world. One aspect of smoking cessation that merits in-depth study is the use of an application designed for smartphones (app), as a supportive element that could assist younger smokers in their efforts to quit. To assess the efficacy of an intervention that includes the assistance of a smoking cessation smartphone application targeted to young people aged 18 to 30 years who are motivated to stop smoking.

**Methods/design:**

Cluster randomised clinical trial. Setting: Primary Health Care centres (PHCCs) in Catalonia. Analyses based on intention to treat. Participants: motivated smokers of 10 or more cigarettes per day, aged 18 to 30 years, consulting PHCCs for any reason and who provide written informed consent to participate in the trial. Intervention group will receive a 6-month smoking cessation programme that implements recommendations of a Clinical Practice Guideline, complemented with a smartphone app designed specifically for this programme. Control group will receive the usual care. The outcome measure will be abstinence at 12 months confirmed by exhaled-air carbon monoxide concentration of at least 10 parts per million at each control test.

**Discussion:**

To our knowledge this is the first randomised controlled trial of a programme comparing the efficacy of usual care with a smoking cessation intervention involving a mobile app. If effective, the modality could offer a universal public health management approach to this common health concern.

**Trial registration:**

NCT01734421

## Background

Tobacco consumption is the most preventable cause of morbidity-mortality in the world. Although the prevalence of tobacco use among adolescents and young adults in Europe has decreased in recent years, tobacco use continues to be a major health problem among young people with a lower educational level. According to, data from ESCA 2011, 30.6% of boys and 34.1% of girls, among 15–24 years smoke daily.

Many adolescent and young adult smokers want to stop smoking and report frequent attempts to quit [1]. Valdivieso et al. [2] recently reported high interest in smoking cessation (67%) among young people who were habitual smokers and who frequently expressed the difficulty of maintaining abstinence despite repeated attempts.

Primary health care has an essential role in smoking cessation. Results of a randomized clinical trial in Spain indicate that a smoking cessation intervention was effective in the adult population, with 8.1% of the intervention group participants reporting continued abstinence at one year, compared to 5.8% in the control group (p<0.014) [3].

However, no studies to date assessed the effectiveness of a structured smoking cessation intervention directed at young people. One aspect of smoking cessation that merits in-depth study is the potential usefulness of new technologies, in particular the use of an application designed for smartphones (app), as a supportive element that could assist younger smokers in their efforts to quit. New technologies –and specifically a targeted app-- offer three benefits that can be useful for this purpose: First, they reach people “where they are”, i.e. there is no need to go to any specific place to receive support services. Second, they promote interactivity, opening new channels of communication between patients and “experts”. And third, they provide instant access to information and assistance. Therefore, it seems logical to hypothesize that a smartphone app will be a useful tool to offer additional support when a young adult wants to stop smoking [4].

Some efforts have already been made in this direction. Clinical trials have evaluated the effectiveness of smoking cessation interventions in adolescents and young people that made use of text messages (SMS), with significantly better results than in the control group (9% vs. 4% abstinence) [5,6]. A recent review by Cochrane [7] of the effectiveness of smoking cessation interventions that use mobile telephones concludes that further studies are needed to determine whether these technologies will assist in smoking cessation efforts. Of particular interest for clinical practice, a programme of text messages sent to mobile phones was effective in the short term (six weeks) and a combined programme of Internet and mobile phone messages up to 12 months.

According to a study by Fundación Telefónica (the foundation associated with Spain’s national telephone company), the number of “smartphones” (mobile phones with an advanced operating system such as Android or iOS) sold in the second trimester of 2011 exceeded for the first time the number of personal computers sold (107 million smartphones, compared to 85 million personal computers). In 2009, more than two thirds of the world’s population had mobile phone access and more than 4.2 billion text messages were sent [4]. We would point out that the youngest segment of the population is most likely to include new technologies in their everyday lives. This behaviour pattern is repeated in patterns of Internet access using mobile phones and 3G technologies, reaching 26% market penetration among young people in Spain --more than twice the European average for this population [4].

Parallel to the growth of smartphone devices, a series of apps have been developed with the objective of providing information and support concerning social and medical problems [8]. The world market for medical apps for smartphones and tablets multiplied seven-fold in 2011 alone, reaching a total of US$718 million, according to a market analysis by the American firm *research2guidance*[9]. At present, these apps are being used as instruments of patient education and support and are also helpful to healthcare professionals [10].

Nonetheless, the market for health care apps is very fragmented; many are designed for very specific contexts or low-incidence diseases. To our knowledge, no randomized studies have assessed the effectiveness of smoking cessation attempts in young people motivated to stop smoking using a smartphone app.

The objective of the present study is to evaluate the efficacy of an intervention that incorporates a smartphone app specifically designed to reduce the prevalence of tobacco consumption in motivated young adults aged 18 to 30 years.

## Methods

### Study design

This project will be developed in two phases, first the design and development of the specific app, followed by a randomized, clustered clinical trial aimed at assessing the efficacy of the smartphone app. The unit of randomization will be the primary care centres of Catalonia, which will assess the effectiveness of the app in achieving smoking cessation among motivated young smokers.

### Participants

#### Selection of participating primary care centres

Participation by primary care centres will be totally voluntary. At the beginning of the study a personalized letter will be sent to the attention of the centre administrators, requesting their collaboration and providing a copy of the study protocol. Within two weeks, the administrators will be contacted by telephone to confirm their participation. Each centre that agrees to participate will be randomized to the intervention or control group and a visit will be scheduled to inform the participating primary care physicians and nurses about the study.

#### Randomisation

The unit of randomisation will be the primary care centre, stratified according to urban/rural location and number of participating professionals to ensure comparability between the study groups, and then assigned 1:1 to the intervention or control group. Randomisation will use the Epidat 3.0 programme.

#### Blinding

Given the nature of the intervention, it is impossible to mask the participants or professionals to the study group assignments. However, data analysis will be blinded to any potential identifiers.

#### Patient inclusion criteria

a) Young adults aged 18 to 30 years who smoke at least 10 cigarettes per day; b) availability of the patient’s clinical history in the primary care centre; c) access to a smartphone device that meets the app requirements; d) access to an Internet data connection; and e) normal to high motivation to stop smoking on the Richmond test (score of 5 or higher).

#### Patient exclusion criteria

a) Addiction to other psychoactive substances; b) any baseline psychotic disease; c) use of a mobile device that does not comply with the app requirements; d) smokers with low motivation to stop smoking on the Richmond test (score of 4 or lower).

### Intervention

**Figure 1:**
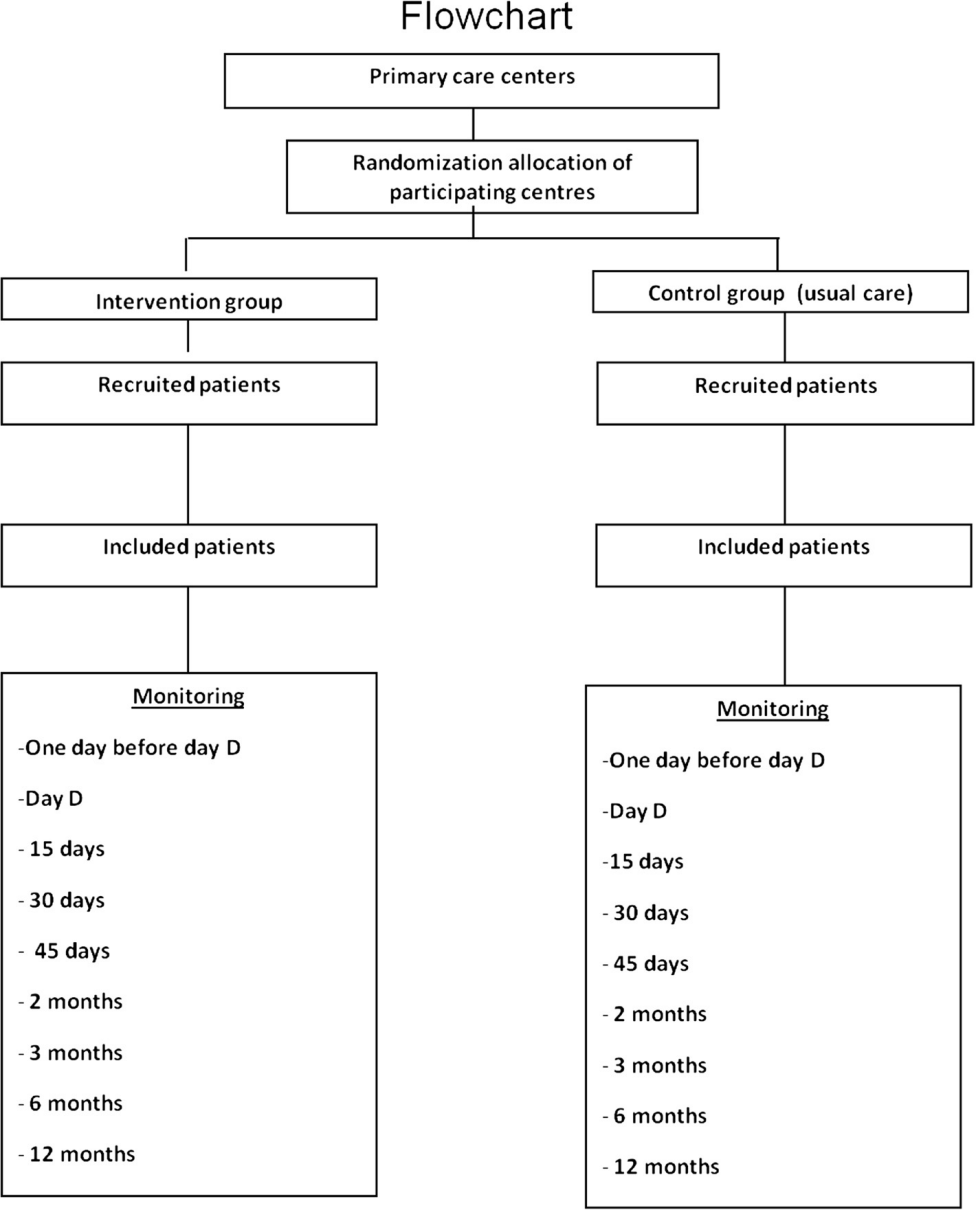
Flowchart: randomisation of centres, and sampling and monitoring of patients.

During the recruitment period, or until the required quota of patients is reached, the family doctor and/or primary care nurse will invite patients aged 18 to 30 years who smoke 10 or more cigarettes per day to participate in the study. If the patient is eligible for inclusion and agrees to participate, informed consent will be requested (Figure 1). After signing informed consent, the participant’s demographic data will be collected and the study questionnaire will be administered (Fageström & Richmond). During this visit (visit 0) smokers with moderate motivation to stop smoking (Richmond score of 5–6) will be invited to schedule a second appointment to attempt to increase their motivation. For highly motivated participants (Richmond score of 7–10) a “quit day” will be established within the following 15 days (D-Day), and they will be invited to schedule an appointment for the day before their D-Day.

At the pre-D-day appointment, smokers will begin their smoking cessation plan according to the clinical practice guidelines of the Catalan Health Institute. Participants randomized to the intervention group will receive the smartphone app, along with an explanation of how to use it to support their smoking cessation plan.

Follow-up and treatment for participants in both the intervention and control groups will follow the clinical practice guidelines. Participants who do not attend follow-up appointments will be telephoned, and will be considered relapsed as of that point.

#### Development of the mobile app

The app we are designing is included in what are called *serious games*, *half games* or *healthy games*. These types of apps have begun to gain recognition as powerful learning tools [8], since they offer those who play the opportunity to develop skills and strategies while they try to achieve the game’s objectives. In contrast to traditional educational methods, serious games are more focused on the views and participation of the learner, rather than the unidirectional guidance of the trainer. A serious game itself will not make smokers quit, but it will help to educate and guide those who wish to end their habit.

The three main objectives for app development are the following: (i) help the user record the progress of the smoking cessation plan; (ii) inform and educate the user about the problems of smoking and the improvements to be anticipated from smoking cessation; (iii) provide alternatives to the user for the times when he or she particularly wants to smoke; for example, a game might provide both entertainment (distraction) and subliminal or indirect messages that can help avoid the urge to smoke. The app will be adapted to the specific stages of the cessation process (typically 6 months duration), and will adjust to the progress of each participant.

To maximize the success of the intervention, a panel of smoking cessation experts, young smokers and ex-smokers, computer programmers, game developers, graphic designers and education experts will be involved at the different stages of the design and development of this app. Although we expect many ideas to emerge during the design process, we envision several options that will be offered to the participant to achieve these objectives:

(1) Information about tobacco: answers to frequently asked questions, advice, suggestions, definitions, illustrations and images.

(2) Private social network for study participants and staff: participants can communicate with each other to ask for help, share concerns, or offer help to others. The project staff will also post information about the process and offer helpful and supportive messages through this network.

(3) Minigame(s): games designed specifically to entertain and educate the participants.

(4) Progress registry: a visual representation of the evolution of the participant’s health progress through the treatment process.

### Control group

Normal practice will be used in counselling controls, following the clinical practice guidelines of the Catalan Health Institute.

### Measures

During the recruitment visit, sociodemographic and anthropometric variables will be recorded: age, sex, educational level, employment, weight, height and blood pressure. Social class will be categorized according to the British Registrar General’s Social Classification [11]. Variables related to tobacco consumption also will be recorded: number of cigarettes smoked per day, start age, number of previous cessation attempts, longest period of cessation, and presence of other smokers in the family. The degree of nicotine dependence will be measured by the Fageström test. Motivation to stop smoking will be assessed by the Richmond test.

During follow-up visits, weight, height, and blood pressure will again be measured. Participants will be asked about their tobacco use and presence of withdrawal symptoms (vehement desire to smoke, anxiety, tension, nervousness, frustration, impatience, difficulty in concentrating, hunger, drowsiness or difficulty in sleeping, nightmares, headache, irritability/depression), and CO in exhaled breath will be measured by carboximetry.

At each visit, the professional who provided smoking cessation information (doctor or nurse) and any treatment provided will be recorded. We will also record the smoking cessation date (D-day) and the date of the end of follow-up, either 12 months after D-day or date lost to follow-up (D2). We will then calculate the length of the follow-up period. (With these variables the length of survival can be calculated: time interval between start date of smoking cessation and relapse or end of follow-up).

Tobacco abstinence will be confirmed by the level of carbon monoxide (CO), set as 10 parts per million or less, measured by carboximetry readings taken by trained personnel.

The follow-up period will have the following end-points: (1) 12 months after D-Day; (2) participant decides not to continue in the study, informs study personnel, and abandons participation; (2) participant is lost to follow-up, provides no more information, and will be considered relapsed as of that time point.

### Sample size/power calculation

To calculate sample size for each primary care centre, the number of individuals in a simple randomized design was multiplied by the design effect. Accepting an alpha risk of 0.05 and a beta risk of 20% in a bilateral contrast, 222 subjects are required in each group to detect a difference of at least 5% (calculations based on EPIDAT v. 3.1). To calculate design effect, we estimated the intracluster correlation coefficient (ICC) in clinical trials randomized by primary care clusters, generally less than 0.05 [12], and as a cluster size. The average size is 20 with a design effect of 1.36. Assuming these values, the definitive sample size for the study was set at 604 subjects, randomised as described above.

### Statistical analysis

Analysis will be performed as intention to treat. At baseline, the comparability of the intervention and control groups will be assessed. Quantitative variables will be described as the mean and standard deviation if they have a normal distribution, or median and range, as appropriate. Qualitative variables will be described as percentages and confidence intervals. Quantitative baseline characteristics will be compared using Student t test. Qualitative variables will use the Pearson chi squared test.

Multi-level multivariate survival analysis will include two effects (fixed and random). The fixed component of the adjusted model will include the group assignment and a dummy variable for baseline and 12-month measurements of the study variables, as well as interactions between relevant variables. All significant covariates in the bivariate analyses (p< 0.30) will be included in the model. This strategy will sufficiently reduce the probability of leaving relevant variables out of the model.

The primary response variable will be continued abstinence at 12 months after beginning smoking cessation (D-Day). We will use multiple techniques to deal with missing values in covariates that are candidates for inclusion in multi-level models. The ’ICE” programme of the STATA-10.1 package estimates missing values by conditional logistic regression, concatenating them with the variables that have complete values. As a convention, a combination of 10 different estimates will be used that includes the variability of these estimates. This will preclude a loss of power due to missing data. In addition, analysis of the models will be repeated to determine sensitivity, using only the subjects for whom complete data are available.

### Ethical aspects of the study

The study will be conducted in accordance with the principles of the Helsinki Declaration, as revised and updated, and will follow Spain’s best practice guidelines (*Buena Práctica Clínica*). The protocol was evaluated by our institute’s ethics committee (*Comité Ético de Investigación Clínica [CEIC], Institut d’Investigació en Atenció Primària [IDIAP] Jordi Gol*). Data confidentiality will be protected under the Spanish law governing the protection of personal data (Ley orgánica de Protección de Datos de Carácter Personal (15/1999 13 December).

## Discussion

The development of the proposed smartphone app, and subsequent evaluation of its effectiveness in our population, has the potential to contribute a useful tool for the smoking cessation efforts of young adults, as well as providing information about the effectiveness of smoking cessation interventions in a population that has not been studied in our environment.

A limitation of the study is its open character. Both patients and health care professionals will know that they are involved in the intervention, which might create a bias. The analysis will be blind, with no identifiers available to the statistician that would indicate whether a subject was assigned to the intervention or control group. The study will use random clustering of primary care centres to avoid the risk of contamination by taking geography into account.

The randomization process is intended to result in a balanced distribution of sociodemographic and cultural characteristics between intervention and control groups, both for doctors and nurses and for participating patients. To avoid contamination between the intervention and control groups, the game and the app itself will only be made available to the intervention group. Multivariate analysis will be used to control for possible confounding factors.

Another limitation is the difficulty of recruiting young patients who want to stop smoking. The multicentre study design, with 22 participating primary care centres and a long (18-month) recruitment period, is intended to ensure an adequate participant pool.

## Competing interests

The authors declare no competing interests.

## Authors’ contributions

EV-L, GF-M, CR-R, JD-G, JD, MLB and AV were responsible for study conceptualization, design, and developing the analytic plan. EV-L and GF-M drafted the manuscript. All authors read and approved the final manuscript.

## Pre-publication history

The pre-publication history for this paper can be accessed here:

http://www.biomedcentral.com/1471-2458/13/704/prepub

## References

[B1] Sussman S Dent CW Nezami E Stacy AW Burton D Flay BR Reasons for quitting and smoking temptation among adolescent smokers: gender differences Subst Use Misuse 1998 33 2703 2720 10.3109/10826089809059346 9869439

[B2] Valdivieso E Rey C Barrera M Arija V Basora J Marsal JR Factors associated with commencing smoking in 12-year-old students in Catalonia (Spain): a cross-sectional population-based study BMC Public Health 2010 10 665 10.1186/1471-2458-10-665 21044344 PMC3091575

[B3] Cabezas C Advani M Puente D Rodriguez-Blanco T Martin C Effectiveness of a stepped primary care smoking cessation intervention: cluster randomized clinical trial (ISTAPS study) Addiction 2011 106 1696 1706 10.1111/j.1360-0443.2011.03491.x 21561497

[B4] Fundación telefónica La sociedad de la información en España 2011 2012 Disponible en: http://e-libros.fundacion.telefonica.com/sie11/

[B5] Free C Whittaker R Knight R Abramsky T Rodgers A Roberts IG Txt2stop: a pilot randomised controlled trial of mobile phone-based smoking cessation support Tob Control 2009 18 88 91 10.1136/tc.2008.026146 19318534

[B6] Free C Knight R Robertson S Whittaker R Edwards P Zhou W Rodgers A Cairns J Kenward MG Roberts I Smoking cessation support delivered via mobile phone text messaging (txt2stop): a single-blind, randomised trial Lancet 2011 378 49 55 10.1016/S0140-6736(11)60701-0 21722952 PMC3143315

[B7] Whittaker R Borland R Bullen C Lin RB McRobbie H Rodgers A Mobile phone-based interventions for smoking cessation Cochrane Database Syst Rev 2009 4 CD006611 10.1002/14651858.CD006611.pub2 19821377

[B8] Stapleton A Serious Games: Serious Opportunities 2004 Melburne: Australian Game Developers' Conference, Academic Summit http://andrewstapleton.com/wp-content/uploads/2006/12/serious_games_agdc2004.pdf

[B9] Jahns R-G Gair G US$ 1.3 billion: The market for mHealth applications in 2012 Disponible en: http://www.research2guidance.com/us-1.3-billion-the-market-for-mhealth-applications-in-2012/.2012

[B10] Acta Sanitaria El mercado de aplicaciones médicas para smartphones se multiplicó por siete en 2011 Disponible en: http://www.actasanitaria.com/areas-sanitarias/tecnologia/articulo-el-mercado-de-aplicaciones-medicas-para-smartphones-se-multiplico-por-siete-en-2011.html

[B11] Domingo-Salvany A Bacigalupe A Carrasco JM Espelt A Ferrando J Borrell C del Grupo de Determinantes Sociales de la Sociedad Española de Epidemiología [Proposals for social class classification based on the Spanish National Classification of Occupations 2011 using neo-Weberian and neo-Marxist approaches] Gac Sanit 2013 27 263 72 10.1016/j.gaceta.2012.12.009 23394892

[B12] Campbell M Grimshaw J Steen N Sample size calculations for cluster randomised trials. Changing Professional Practice in Europe Group (EU BIOMED II Concerted Action) J Health Serv Res Policy 2000 5 12 16 10787581 10.1177/135581960000500105

